# SLC25A28 Overexpression Promotes Adipogenesis by Reducing ATGL

**DOI:** 10.1155/2024/5511454

**Published:** 2024-05-04

**Authors:** Hua Guan, Lin Xiao, Kaikai Hao, Qiang Zhang, Dongliang Wu, Zhanyi Geng, Bowen Duan, Hui Dai, Ruifen Xu, Xuyang Feng

**Affiliations:** ^1^Shaanxi Key Laboratory of Ischemic Cardiovascular Diseases & Institute of Basic and Translational Medicine, Xi'an Medical University, Xi'an 710021, Shaanxi, China; ^2^Department of Cardiology, Xijing Hospital, The Fourth Military Medical University, Xi'an 710032, China; ^3^Department of Cardiology, Xianyang Hospital of Yan'an University, Xianyang 712000, China; ^4^Department of Clinical Medicine, Gansu Medical College, Pingliang 744000, China; ^5^Department of Anesthesiology, Shaanxi Provincial Peoples Hospital, Xi'an 710068, China; ^6^Department of Neurology, Xianyang Hospital of Yan'an University, Xianyang 712000, China

**Keywords:** adipose tissue, lipid accumulation, obesity, SLC25A28/mitoferrin 2

## Abstract

Adipose tissue dysfunction is seen among obese and type 2 diabetic individuals. Adipocyte proliferation and hypertrophy are the root causes of adipose tissue expansion. Solute carrier family 25 member 28 (SLC25A28) is an iron transporter in the inner mitochondrial membrane. This study is aimed at validating the involvement of SLC25A28 in adipose accumulation by tail vein injection of adenovirus (Ad)-SLC25A28 and Ad-green fluorescent protein viral particles into C57BL/6J mice. After 16 weeks, the body weight of the mice was measured. Subsequently, morphological analysis was performed to establish a high-fat diet (HFD)-induced model. SLC25A28 overexpression accelerated lipid accumulation in white and brown adipose tissue (BAT), enhanced body weight, reduced serum triglyceride (TG), and impaired serum glucose tolerance. The protein expression level of lipogenesis, lipolysis, and serum adipose secretion hormone was evaluated by western blotting. The results showed that adipose TG lipase (ATGL) protein expression was reduced significantly in white and BAT after overexpression SLC25A28 compared to the control group. Moreover, SLC25A28 overexpression inhibited the BAT formation by downregulating UCP-1 and the mitochondrial biosynthesis marker PGC-1*α*. Serum adiponectin protein expression was unregulated, which was consistent with the expression in inguinal white adipose tissue (iWAT). Remarkably, serum fibroblast growth factor (FGF21) protein expression was negatively related to the expansion of adipose tissue after administrated by Ad-SLC25A28. Data from the current study indicate that SLC25A28 overexpression promotes diet-induced obesity and accelerates lipid accumulation by regulating hormone secretion and inhibiting lipolysis in adipose tissue.

## 1. Introduction

The most recent World Health Organization (WHO) statistics indicate that obesity is a prevalent global health concern, with over 2 billion adults (aged 18 and over) worldwide developing overweight, and among them, over 600 million adults are obese [1]. Obesity is a serious metabolic disease that increases the risk of various diseases, including but not limited to the following: leading to the occurrence of cardiovascular diseases specifically atherosclerosis, hypertension, coronary heart disease, and stroke [2]. Long-term obesity induces low-grade systemic inflammation and insulin resistance [3]. Obesity can lead to respiratory system diseases, especially sleep apnea syndrome; patients will repeatedly experience breathing pauses during sleep, leading to a decrease in sleep quality [4]. Obesity can lead to nonalcoholic fatty liver disease, and severe cases can develop into liver cirrhosis [5]. Obesity is also associated with the occurrence of many cancers, such as ovarian, breast, endometrial, colon, and prostate cancers [6]. Globally, the health and economic costs caused by obesity have become a serious public health problem [7].

Adipose tissue contains a large number of fat cells and is among the most important metabolic organs in the human body [8]. Its morphological features include single or aggregated spherical or hemispherical fat masses, surrounded by a certain number of blood vessels, nerves, and connective tissue support [9]. The two categories of adipose tissue include brown adipose tissue (BAT) and white adipose tissue (WAT). Adult humans, newborns, and other mammals contain BATs in the body regions such as the neck, abdomen, chest, and scapula, including a large number of mitochondria and brown adipose bodies on the inner membrane [10]. BAT activation is triggered by exposure to cold, commencing a process termed nonshivering thermogenesis, which entails dissipation of energy as heat. The aforementioned process is mediated by uncoupling protein-1 (UCP-1) [11], which is located at the inner mitochondrial membrane of brown adipocytes, where it functions to uncouple chemical energy [12]. Concerning its role in enhancing energy expenditure, there exists an inverse correlation between BAT mass and activity with obesity and body mass index [13]. WAT is distributed throughout the body and is mainly responsible for storing thermal energy and nutrients, playing an important role in energy storage and regulation and participating in the regulation of inflammatory responses, secretion of metabolic substances, and neural regulation [14]. In summary, adipose tissue has an essential regulatory function in the human body, and changes in its morphology and function are closely related to many diseases such as obesity, metabolic syndrome, and cardiovascular disease [15].

The solute carrier family 25 member 28 (SLC25A28), also denoted as mitochondrial ferritin 2, is an inner membrane protein located on human chromosome 22q12.3 [16]. This amino acid residue has two transmembrane domains and a mitochondrial targeting sequence [17]. SLC25A28 plays a key role in iron transportation and storage. As an iron carrier in mitochondria, SLC25A28 transports free iron ions from the cytosol to mitochondria to support iron-dependent reactions and oxidative phosphorylation [18]. In addition, SLC25A28 regulates the permeability of mitochondrial membranes and the form of fixed iron to maintain the balance and stability of iron within mitochondria [19]. Abnormal function of SLC25A28 may lead to iron metabolism disorders and mitochondrial dysfunction, which are related to various diseases such as anemia [20], heart disease [21], and cancer [22].

In the past decade, SLC25A28 has received more and more attention due to its important role in iron transport [23]. It was reported that SLC25A28/37 double-deficiency attenuated the expression of adipogenic genes and synthesis of lipids during adipogenic differentiation in 3T3-L1 cells, but its effect on adipogenesis and lipid metabolism in mice is unknown. Using the adenovirus overexpression technique, we analyzed the impacts of overexpression of SLC25A28 on lipid metabolism in the circumstances of high-fat diet (HFD).

## 2. Methods

### 2.1. Animals

Eight-week-old male mice have a weight ranging between 20 and 22 g (six mice in each group). All animals are housed in an air-conditioned room, while the room temperature can be maintained at 20~26°C and a relative humidity of 50%–80% under a 12-h light and 12-h dark cycle according to the SPF Animal Room Temperature and Humidity Standards.

**Figure 1:**
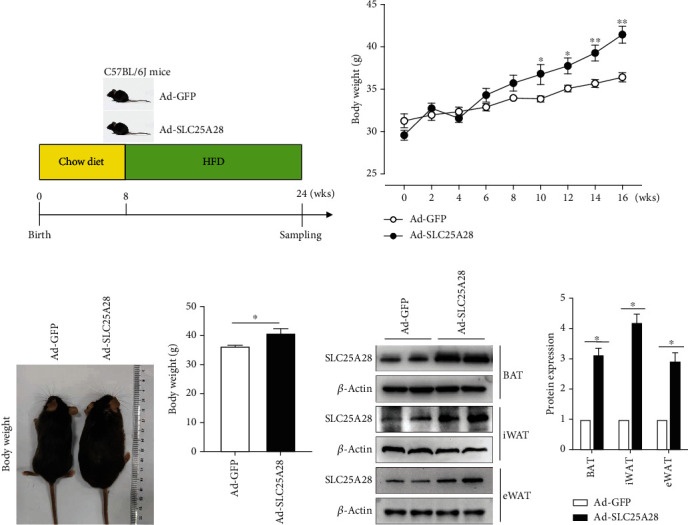
The experimental flow chart and body weight. (a) Male, 8-week-old mice, weighing 20–22 g. The animals were randomly divided into two groups: adenovirus-green fluorescent protein (Ad-GFP) or Ad-SLC25A28. 1 × 10^11^ viral particles of Ad-SLC25A28 or Ad-GFP were injected into the tail vain of mice. Ad-GFP or Ad-SLC25A28 were administered every 3 weeks and injected six times in total. After the injection of adenovirus, all the mice were fed with a high-fat diet (HFD). (b) The body weight of mice (mean ± SEM; *n* = 6 for each group, ANOVA, ^∗^*p* < 0.05, ^∗∗^*p* < 0.01). (c) Comparison of body shape and (d) weight of mice after 16 weeks of HFD feeding (mean ± SEM; *n* = 6 for each group, *t*-test, ^∗^*p* < 0.05). (e) Protein expression of SLC25A28 in eWAT, iWAT, and BAT was determined by western blotting (mean ± SEM; *n* = 6 for each group; *t*-test, ^∗^*p* < 0.05). (f) Quantification of western blot analysis (*β*-actin as the loading control). Results are expressed as fold compared with the Ad-GFP group (mean ± SEM; *n* = 6 for each group; *t*-test, ^∗^*P* < 0.05, Ad-SLC25A28 vs. Ad-GFP). Ad-GFP: adenovirus-green fluorescent protein; BAT: brown adipose tissue; high-fat diet: HFD; SLC25A28: solute carrier family 25 member 28.

Random grouping of the mice established two groups: adenovirus-green fluorescent protein (Ad-GFP) or Ad-SLC25A28 were administered six times in total every 3 weeks. After the injection of adenovirus, all the mice were fed with HFD. The experimental flow chart is shown in Figure 1(a).

Euthanasia was induced by intraperitoneal injection of pentobarbital sodium (150 mg/kg). The criteria for confirmation of death included absence of blink reflex and lack of spontaneous breathing for 2–3 min. The animal experiments were executed as per the protocols for animal experiments of Xi'an Medical University, which was adapted from the Guide for the Care and Use of Laboratory Animals (NIH Publication, 8th Edition, 2011). The Laboratory Animal Administration Committee of Xi'an Medical University granted its approval to all animal experiments.

### 2.2. Creation of the Adenoviral SLC25A28 Vector and Cell Infection

A recombinant adenoviral vector encoding SLC25A28 (Ad-SLC25A28) was created as per the previously documented method [24]. After purification was accomplished, the viral titer was examined by TCID50. A control was established by constructing an empty adenoviral vector (Ad-GFP).

### 2.3. Plasma Lipid Test

The protocol was performed as previously published [25]. High-density lipoprotein cholesterol (HDL-C), low-density lipoprotein cholesterol (LDL-C), triglyceride (TG), and plasma total cholesterol (TC) were examined with the aid of commercial assay kits (Biosino Bio-Technology & Science Inc.) [26]. Plasma free glycerol was detected with assay kit (Beyotime Biological Technology Co., Ltd.). Plasma adipose triglyceride lipase (ATGL) protein expression was measured by ELISA kit (Shanghai Jingkang Biotechnology Co., Ltd.).

### 2.4. Glucose Tolerance Test (GTT)

According to previous publications [27], GTT was executed on 4–6 h fasted mice. Mice were intraperitoneally injected (i.p.) with 25% (w/v) glucose (1.5 g glucose/kg BW). Glycemic levels for GTT were measured before administration as well as 15, 30, 60, 90, and 120 minutes post glucose challenge utilizing OneTouch® Glucometer (New Brunswick).

### 2.5. Morphology Analysis of Adipose Tissue

Excision and fixation of adipose tissues in 10% formalin buffer were performed according to the protocol published [28]. We isolated the BAT from the triangular area of the scapula for subsequent analysis. Furthermore, fixed specimens were processed to frozen blocks, sectioned, and stained with hematoxylin-eosin (H&E) as per standard procedures. A microscope containing a digital camera (Nikon, Tokyo, Japan) was utilized to photograph the sections for microscopic quantification. Additionally, ImageJ software measured both the diameter and area of adipocytes.

### 2.6. Western Blot Analysis

Western blot analysis was performed as previously described [29]. Primary antibodies included SLC25A28 (Abcam), adiponectin (R&D), fibroblast growth factor 21 (FGF21) (R&D), fatty acid binding protein 4 (FABP4) (R&D), UCP-1 (R&D), peroxisome proliferator-activated receptor gamma coactivator 1-alpha (PGC-1*α*) (Abways), ATGL (Abmart), perilipin 1 (Abways), perilipin 2 (Abways), leptin (R&D), and *β*-actin (Santa Cruz).

### 2.7. Statistical Analysis

All data were presented as the mean ± SEM. Statistical analyses were executed through either Student's *t*-test with an equal *F* value or Welch's *t*-test in circumstances where the *F-*value was unequal. Two-way ANOVA ascertained the differences between different groups in the same treatment. *P* < 0.05 denoted statistical significance. Furthermore, GraphPad Prism 5.0 (GraphPad Software, Inc., San Diego, CA, USA) executed all analyses.

## 3. Results

### 3.1. SLC25A28 Overexpression Promoted Weight Gain in Mice

To evaluate the impact of SLC25A28 on the adipose tissue lipid accumulation induced by HFD, we established an animal model by tail vein injection (Figure 1(a)). Ad-SLC25A28 or Ad-GFP virus particles were injected separately to observe the variety of body weight. The result showed that the body weight was increased remarkably in the Ad-SLC25A28 group in contrast to the control group (Figures 1(b)–1(d)). We tested the protein expression level of SLC25A28 in inguinal adipose tissue (iWAT), epididymal adipose tissue (eWAT), and BAT after being administrated by tail vein injection of Ad-SLC25A28 or Ad-GFP. SLC25A28 acts as a mitochondrial iron transporter that mediates the uptake of iron. Additionally, SLC25A28 is likely involved in the synthesis of heme in hemoproteins and Fe-S cluster assembly in nonerythroid cells. Brown adipocytes are rich in mitochondria, and cell sublocalization analysis shows that SLC25A28 has the highest expression in mitochondria and lower expression levels in other organelles or cytosol. Therefore, western blotting established that the protein expression level of SLC25A28 in BAT was elevated ~1.5 fold after administration with Ad-SLC25A28 (Figures 1(e) and 1(f)).

### 3.2. Effects of SLC25A28 Overexpression on Blood Glucose and Lipids in Mice

**Figure 2:**
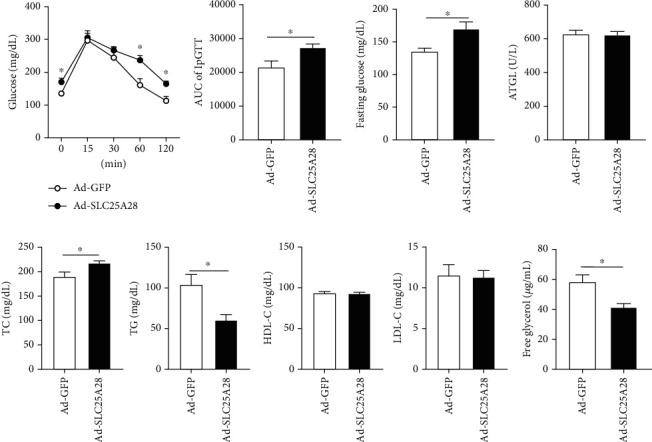
The overexpression of SLC25A28 reduced glucose clearance efficiency when following a HFD. (a) IpGTT in mice with 12 weeks of HFD (mean ± SEM; *n* = 6 for each group; ANOVA, ^∗^*p* < 0.05). (b) Bar graphs are the AUC of ipGTT calculated from the original graph (mean ± SEM; *n* = 6 for each group; *t*-test, ^∗^*p* < 0.05). (c) Fasting blood glucose concentration was measured after feeding for 12 weeks of HFD (mean ± SEM; *n* = 6 for each group; *t*-test, ^∗^*p* < 0.05). (d) Plasma ATGL activity was measured by Elisa kit (mean ± SEM; *n* = 6 for each group; *t*-test). (e) Plasma TC, TG, HDL-C, and LDL-C were measured (mean ± SEM; *n* = 6 for each group; *t*-test, ^∗^*p* < 0.05). (f) Plasma free glycerol levels in plasma were measured (mean ± SEM; *n* = 6 for each group; *t*-test, ^∗^*p* < 0.05). Ad-SLC25A28 versus Ad-GFP. AUC: the area under the curve; ATGL: adipose triglyceride lipase; FFA: free fatty acids; IpGTT: intraperitoneal glucose tolerance test; TC: total cholesterol; TG: triglyceride; HDL-C: high-density lipoprotein cholesterol; LDL-C: low-density lipoprotein cholesterol.

To ascertain the effect of SLC25A28 on blood glucose, GTT was conducted to establish the glucose level at 15, 30, 60, and 120 min. In comparison to the control group, glucose clearance efficiency decreased significantly in the Ad-SLC25A28 group (Figures 2(a) and 2(b)). Additionally, SLC25A28 overexpression increased the fasting glucose compared to the GFP group (Figure 2(c)). Otherwise, the results showed that there were no changes in plasma ATGL enzyme products after overexpression of SLC25A28 compared to the GFP group (Figure 2(d)). Additionally, there was a significant reduction of free glycerol in the Ad-SLC25A28 group compared to the control group (Figure 2(f)). Moreover, after overexpression of SLC25A28, plasma TC was increased significantly, while plasma TG was decreased substantially compared to the GFP group (Figure 2(e)). HDL-C and LDL-C have no remarkable change between Ad-SLC25A28 and Ad-GFP groups (Figure 2(e)).

### 3.3. SLC25A28 Overexpression Promoted Lipid Accumulation in Mice

**Figure 3:**
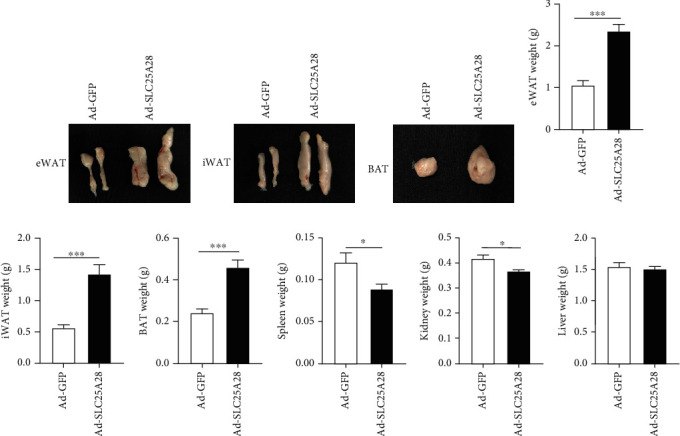
SLC25A28 promoted the lipid accumulation induced by HFD. (a–f) Morphological comparison and weight statistics of iWAT, eWAT, and BAT after sacrifice of mice (mean ± SEM; *n* = 6 for each group; *t*-test, ^∗∗∗^*P* < 0.001; Ad-SLC25A28 vs. Ad-GFP). (g–i) The weight of the spleen, kidney, and liver was calculated after the sacrifice of mice (mean ± SEM; *n* = 6 for each group; *t*-test, ^∗^*p* < 0.05; Ad-SLC25A28 vs. Ad-GFP). iWAT: inguinal white adipose tissue; eWAT: epididymal white adipose tissue.

**Figure 4:**
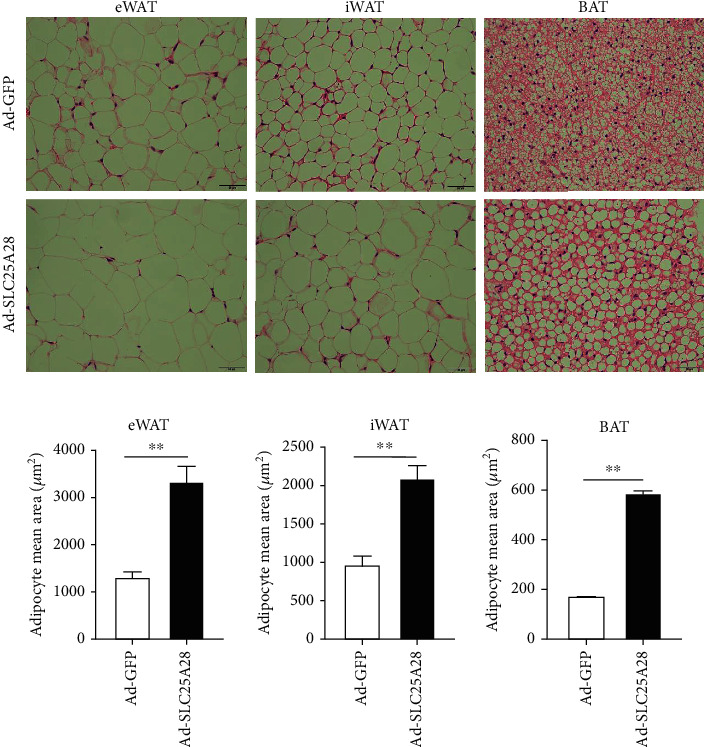
Morphological observation of adipocytes in adipose tissue. (a) Representative image of H&E-stained sections of eWAT, iWAT, and BAT tissues from Ad-SLC25A28 or Ad-GFP group mice with HFD diet. Scale bar: 50 *μ*m. (b) Adipocyte area statistics of eWAT, iWAT, and BAT from HFD-fed mice (mean ± SEM; *n* = 6 of each group; *t*-test, ^∗∗^*P* < 0.01; Ad-SLC25A28 vs. the Ad-GFP group).

To establish what leads to weight gain in mice, we isolated iWAT, eWAT, and BAT of mice, and the findings inferred that compared to the GFP control group, the weight of eWAT, iWAT, and BAT were all increased significantly in Ad-SLC25A28 group (Figures 3(a)–3(f)). However, the weight of the spleen and kidney was lost significantly after overexpression SLC25A28 (Figures 3(g) and 3(h)). Moreover, the liver weight showed no change between the GFP and SLC25A28 groups (Figure 3(i)). H&E staining results of pathological sections of iWAT, eWAT, and BAT revealed that after administration with Ad-SLC25A28, the lipid droplets were greater than that in the control group (Figures 4(a) and 4(b)).

### 3.4. SLC25A28 Overexpression Resulted in Increased Lipogenesis in eWAT, iWAT, and BAT

**Figure 5:**
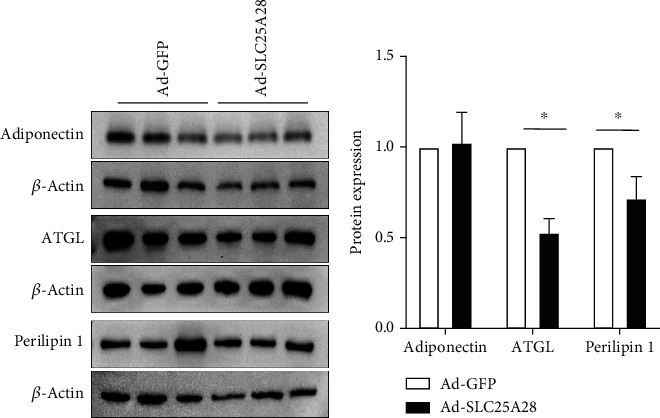
Identify the protein expression level of SLC25A28 in eWAT. (a) Adiponectin, ATGL, and perilipin 1 protein expression in eWAT were measured by western blotting. (b) Quantification of western blotting analysis (*β*-actin as the loading control). Results are expressed as fold compared with the Ad-GFP group (mean ± SEM; *n* = 6 for each group; *t*-test, ^∗^*p* < 0.05 vs. Ad-GFP).

**Figure 6:**
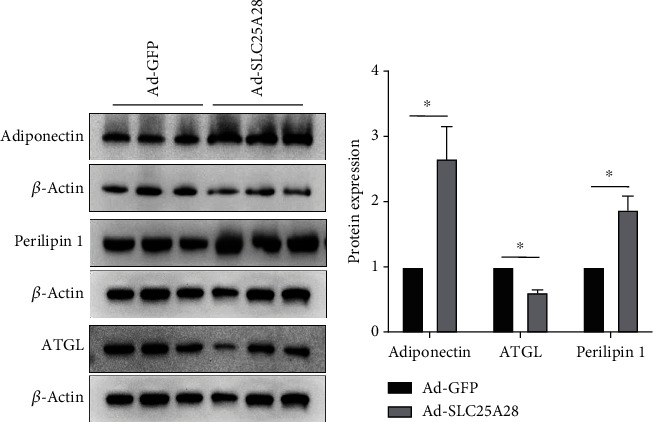
Identify the protein expression level of SLC25A28 in iWAT. (a) Adiponectin, ATGL, and perilipin1 protein expression in iWAT were measured by western blotting. (b) Quantification of western blotting analysis (*β*-actin as the loading control). Results are expressed as fold compared with the Ad-GFP group (mean ± SEM; *n* = 6 for each group; *t*-test, ^∗^*p* < 0.05 vs. Ad-GFP).

**Figure 7:**
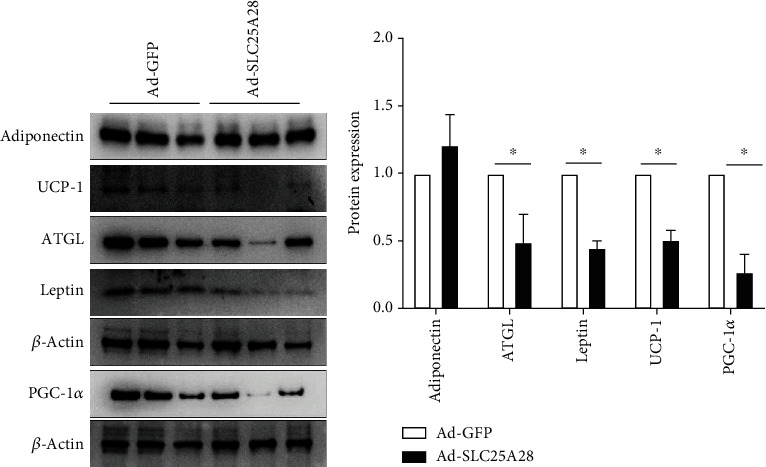
Identify the protein expression level of SLC25A28 in BAT. (a) SLC25A28 regulates adipogenic protein expression in BAT. Adiponectin, ATGL, leptin, UCP-1, and PGC-1*α* protein expression were measured by western blotting. (b) Quantification of western blotting analysis (*β*-actin as the loading control). Results are expressed as fold compared with the Ad-GFP group (mean ± SEM; *n* = 6 for each group; *t*-test, ^∗^*p* < 0.05 vs. Ad-GFP). UCP-1: uncoupling protein 1; PGC-1*α*: peroxisome proliferator-activated receptor *γ*, coactivator 1*α*.

To explore the molecular mechanism of overexpression SLC25A28 promoted accumulation of the content of lipid droplets in WAT and BAT, we isolated the adipose tissue and performed western blotting to identify the lipid synthesis as well as lipolysis marker protein expression. In eWAT, the expression level of lipolysis-related protein ATGL and perilipin 1 decreased significantly after Ad-SLC25A28 was administered (Figures 5(a) and 5(b)). However, adiponectin protein expression showed no change in Ad-SLC25A28 compared to the control group (Figures 5(a) and 5(b)). In iWAT, the lipogenesis-related protein adiponectin and perilipin 1 protein expression level were also elevated significantly after administrated of SLC25A28 compared to the control group; nevertheless, ATGL expression level was decreased significantly in the Ad-SLC25A28 group (Figures 6(a) and 6(b)). In BAT, leptin, UCP-1, and PGC-1*α* protein were all reduced significantly after administrated of SLC25A28 compared to the control group (Figures 7(a) and 7(b)). However, the protein expression of adiponectin was not changed anymore in the Ad-SLC25A28 group (Figures 7(a) and 7(b)).

### 3.5. SLC25a28 Overexpression on the Synthesis of Plasma Adipokines in HFD-Induced Mice

**Figure 8:**
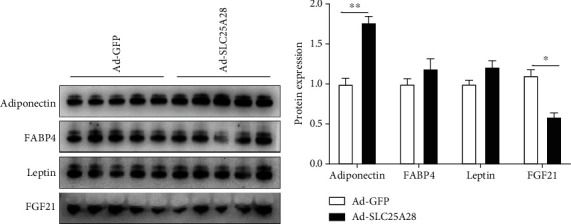
Identify the plasma adipokines in HFD-induced mice. (a) Plasma adiponectin, leptin, FABP4, and FGF21 protein expression were measured by western blotting of Ad-SLC25A28 and Ad-GFP group mice. (b) Quantification of western blotting analysis. Results are expressed as fold compared with the Ad-GFP group (mean ± SEM; *n* = 5 for each group; *t*-test, ^∗^*p* < 0.05 vs. Ad-GFP, ^∗∗^*P* < 0.01). FABP4: fatty acid binding protein 4; FGF21: fibroblast growth factor.

To ascertain the molecular mechanism of overexpression SLC25A28 on the adipocyte-related hormone secretion, we performed western blotting to identify the protein expression of adiponectin, FGF21, leptin, and FABP4 in plasma. Remarkably, plasma FGF21 protein expression was reduced significantly, while plasma adiponectin protein expression was enhanced significantly after administration with Ad-SLC25A28 compared to the GFP group (Figure 8). However, plasma leptin and FABP4 protein expression showed no change in Ad-SLC25A28 compared to the control group (Figure 8).

## 4. Discussion

In our study, we used adenovirus injection to overexpression SLC25A28 whole body wide and affirmed a promoting obesity function of SLC25A28 in diet-induced obesity as well as glucose intolerance impairment. Our findings affirm that SLC25A28 overexpression contributes to increased weight gain and accelerates the occurrence of obesity, as mice with SLC25A28 overexpression manifested an increase in adipocyte size and serum adiponectin, while serum FGF21 decreased. Adiponectin, an adipose cell-synthesized hormone, regulates insulin sensitivity and has functions in obesity [29], diabetes [30], inflammation [31], atherosclerosis, and cardiovascular disease [32]. Increased levels of adiponectin have been affirmed to be linked to the enlargement of adipose tissue [33]. In the present study, serum adiponectin levels of SLC25A28 overexpression mice were unregulated substantially than control mice induced by HFD, which is comprehensible when taking into account their adiposity. Nonetheless, they displayed greater plasma glucose levels. Importantly, this difference in secretion of serum adiponectin is negatively correlated with glucose levels, which is inconsistent with previous reports. It is worth mentioning that adipose mass has a function in plasma adiponectin concentrations, and visceral adipose tissue mass is negatively linked to plasma adiponectin concentrations [34]. Nevertheless, subcutaneous adipose tissue is positively linked to plasma adiponectin concentrations [35]. Hence, future studies should delve into the mechanisms via which SLC25A28 modulates adiponectin synthesis and the subsequent impacts on energy metabolism.

In line with the current experimental results, various studies posit that a decrease in FGF21 results in obesity [36, 37]. Furthermore, serum FGF21 enhances energy homeostasis via distinct metabolic functions in various target organs [38], including modulation of liver fatty acid oxidation [39], causing metabolism of glucose and browning in susceptible WAT depots [40, 41], and playing an anti-inflammatory function in pancreases and cardiac muscle [42, 43]. This suggests a new therapeutic approach for metabolic complications, including fatty liver disease and diabetes [44, 45]. This infers that impaired insulin sensitivity by SLC25A28 overexpression could be mediated by decreases in serum FGF21secretion. It is intriguing to hypothesize that the physiological response mediated by FGF21 remarkably elevates PGC-1*α* protein levels independently of mRNA levels in *vivo* and in *vitro* [46]. Conversely, fat-specific PGC-1*α* knockout mice exhibit an impaired response to FGF21 [46], which was consistent with our results established in the BAT after overexpression SLC25A28. Previous studies demonstrated that in amino acid restriction and impaired hepatic and muscular autophagy animal model [47], enhanced circulating FGF21 elevated UCP-1 expression, suggesting that FGF21 plays a role in browning WAT to adaptive response to cold, which was consistent with our research that decreasing circulating FGF21 induced lowered UCP-1 expression in BAT after overexpression SLC25A28 compared to the control group. When analyzing the experimental results, we also noticed the wonderful changes between the expression level of UCP-1 protein and the amount of BAT between the Ad-GFP and Ad-SLC25A28 administration groups. However, the existing experimental results cannot clearly explain the relationship between them. Normalizing UCP1 chemiluminescence to the micrograms of protein on the gel and accounting for differences in the depot could provide insight into whether the total UCP1 amount changed in BAT or not. The UCP1 protein may be distributed more across the larger tissue.

Besides modulating body weight and serum hormones, overexpression SLC25A28 enhances adipocyte hypertrophy and lipid droplet accumulation, which was exhibited that adipocyte size is larger in iWAT, eWAT, and BAT of overexpression SLC25A28 mice in contrast to control mice. Remarkably, impaired mobilization of TG from adipocytes is expected to lead to obesity and worsen it during nutritional stress [48]. Previous studies have reported that adipocyte or BAT-specific ATGL knockout mice suffer from aggravated obesity induced by HFD, including reduced serum lipids, adipocyte lipolysis, and systemic lipid oxidation [49–52]. Accordingly, this study showed that ATGL protein expression level was lower in WAT and BAT, while the plasma level of TG was reduced, which is specifically observed after systemic overexpression SLC25A28, suggesting that SLC25A28 regulates ATGL protein expression and posttranslational modification, thus affecting diet-induced obesity in mice. In the present experiment, we discovered that perilipin 1 was downregulated in eWAT while unregulated in iWAT. The differential expression of perilipin 1 in eWAT and iWAT adipose tissues may be attributed to the following reasons: First, eWAT primarily participates in energy metabolism and hormone production, while iWAT functions mainly in energy storage and insulation. Second, eWAT has smaller and less stable lipid droplets, while iWAT has larger and more stable lipid droplets, potentially necessitating increased perilipin 1 expression to maintain droplet stability. Therefore, the differential expression of perilipin 1 needs to be defined further.

Furthermore, a previous report infers that SLC25A28 mediates ferrous iron transport across the mitochondrial inner membrane, in nonerythroid cells, and maintains the level of cellular mitochondrial iron [19]. Previous research has also affirmed that knockdown of SLC25A28 decreased the production of reactive oxygen species in osteosarcoma [22]. Therefore, the brown adipocyte is typically manifested by abundant mitochondria and remarkable expression of mitochondrial marker genes. Overexpression SLC25A28 could downregulate the expression of UCP-1 and PGC-1*α*, which stimulated mitochondrial biogenesis as well as function as affirmed by our study. Enhanced production of reactive oxygen species elevates oxidative levels, acts as a damaging mechanism, and serves as a compensatory mechanism for thermogenesis activation.

In the present experiment, we performed GTT to evaluate the glucose metabolism, and the results affirmed that the basal glycemic level was elevated in the Ad-SLC25A28 group compared to Ad-GFP in Figure 2(a). Previous studies have demonstrated that the decreasing sensitivity of insulin in the liver leads to a weakened inhibitory effect on liver-derived gluconeogenesis, resulting in increased gluconeogenesis and elevated blood glucose levels [53–55]. The liver is important in the digestion, absorption, transportation, lipolysis, and synthesis of lipids [56]. Although in this study, we centered on the involvement of lipid metabolism in adipose tissue; otherwise, we found that overexpression of SLC25A28 resulted in elevated basal blood glucose, indicating that SLC25A28 affects blood glucose partly by regulating liver lipid metabolism. Therefore, we will delve into the effects of SLC25A28 in the liver to gain a deeper understanding of lipid metabolism in subsequent experiments.

The BAT in the scapula of the mouse was wrapped in a large amount of WAT when euthanized (Figure 3(c)) [57]. Morphological observation from tissue sections revealed that the diameter of brown adipocytes was substantially elevated following overexpression SLC25A28 compared to the GFP group (Figures 4(a) and 4(b)). Additionally, the SLC25A28 clearly increased the unilocular adipocyte number present in BAT, which was consistent with the general morphology of BAT in Figure 3(c). Previous studies have demonstrated that increasing the volume of BAT can accelerate energy consumption, while on the contrary, slowing down energy metabolism can lead to obesity [58]. Based on the above results, we speculate that one of the explanations for obesity in mice may be that the overexpression of SLC25A28 promotes the transformation of BAT into WAT. Based on the reviewer's valuable suggestions, we will perform further in-depth research on the relationship between energy metabolism and lipid droplet accumulation in adipocytes of HFD-induced SLC25A28 overexpression mice in the future. It is important to explore the relationship between the SLC25A28 and calorie consumption including oxygen consumption and carbon dioxide release in the subsequent experiment.

## 5. Conclusion

In summary, this study demonstrated that overexpression of SLC25A28 promotes lipid accumulation and adipogenesis in mice, impaired glucose tolerance, and exacerbated diet-induced obesity. These findings imply that SLC25A28 plays a modulatory function in the adipocyte hypertrophy phenotype induction, thereby displaying possible therapeutic significance in obesity treatment and glucose metabolism.

## Data Availability

The datasets used and/or analyzed during the current study are available from the corresponding author upon reasonable request.

## Abbreviations

Ad: Adenovirus
ATGL: Adipose triglyceride lipase
BAT: Brown adipose tissue
eWAT: Epididymal adipose tissue
FABP4: Fatty acid binding protein 4
FGF21: Fibroblast growth factor 21
GTT: Glucose tolerance test
GFP: Green fluorescent protein
H&E: Hematoxylin-eosin
HDL-C: High-density lipoprotein cholesterol
HFD: High-fat diet
iWAT: Inguinal white adipose tissue
LDL-C: Low-density lipoprotein cholesterol
PGC-1*α*: Peroxisome proliferator-activated receptor gamma coactivator 1-alpha
SPF: Specific pathogen-free
SLC25A28: Solute carrier family 25 member 28
TC: Total cholesterol
TG: Triglyceride
UCP-1: Uncoupled protein 1
WAT: White adipose tissue
WHO: World Health Organization.
